# Inflammatory and Hematologic Liver and Platelet (HALP) Scores in Hypothermia-Treated Hypoxic–Ischemic Encephalopathy (HIE)

**DOI:** 10.3390/children11010072

**Published:** 2024-01-08

**Authors:** Handan Hakyemez Toptan, Kübra Gökçe Tezel, Oğuzhan Tezel, Ömer Ataç, Gonca Vardar, Sinem Gülcan Kersin, Eren Özek

**Affiliations:** 1 Department of Pediatrics, Division of Neonatology, School of Medicine, Marmara University, Istanbul 34722, Turkey; kubragokce1993@gmail.com (K.G.T.); oguzhantezel92@gmail.com (O.T.); gncvrd14@gmail.com (G.V.); sinemgulcankersin@gmail.com (S.G.K.); ozekeren@gmail.com (E.Ö.); 2 Department of Public Health, International School of Medicine, Istanbul Medipol University, Istanbul 34810, Turkey; oatac@medipol.edu.tr

**Keywords:** hypoxic–ischemic encephalopathy, systemic inflammatory indices, HALP score, neonatal care, hypothermia therapy

## Abstract

Objective: This study examined systemic inflammatory indices and “Hemoglobin, Albumin, Lymphocyte, Platelet (HALP) scores” in neonates with hypoxic–ischemic encephalopathy (HIE). Methods: A total of 43 neonates with moderate-to-severe HIE at 36 weeks’ gestation were assessed. Systemic inflammatory markers were measured before HT commenced within 0–6 h after birth and between 60 and 72 h during and after therapy or before adjusting for hypothermia. Results: Platelet counts, hemoglobin levels, and platelet indices in the HIE group were significantly lower at both time points (*p* = 0.001). Both the neutrophil-to-lymphocyte ratio (NLR) and monocyte-to-lymphocyte ratio (MLR) decreased in the HIE group after hypothermia therapy (*p* = 0.001). Seizures, PVL, and kidney injuries were associated with higher HALP scores. The AUCs of NLR, PLR, MLR, SII, SIRI, and platelet, neutrophil, monocyte, and lymphocyte Index (PIV) showed significant sensitivity and specified HIE, with area under the curve (AUC) values of 0.654, 0.751, 0.766, 0.700, 0.722, and 0.749, respectively. Conclusions: A significant difference in systemic inflammatory markers was found between the HIE and control groups after hypothermia treatment, with significant reductions in the MLR and NLR. These markers, particularly MLR, were significant predictors of adverse clinical outcomes including seizures, PVL, and kidney damage.

## 1. Introduction

Hypoxic–ischemic encephalopathy (HIE) is a critical condition arising predominantly from intrapartum hypoxia, a medically unpredictable and emergent condition that often heralds a range of neurosensory and cognitive deficiencies, profoundly affecting survivors’ quality of life [1]. Given the widespread implications of HIE, it is important to understand its prevalent circumstances and consequences, emphasizing the need for efficient diagnostic and prognostic tools [2]. Approximately 0.75 million infants globally experience moderate-to-severe HIE annually, leading to neurodevelopmental impairment in approximately 400,000 babies [3,4].

The process of identifying infants susceptible to adverse outcomes traditionally involves detailed analysis of the neonatal trajectory enriched by intricate neurological evaluations [2].

Recent advances in neonatology and neurology have illuminated the role of inflammation as an ancillary, yet potent, instigator of neonatal brain injuries in the context of perinatal distress. Inflammatory cascades following hypoxic injury led to the activation of resident and recruited immune cells and the synthesis of cytokines [5,6,7]. Neuroinflammation, marked by the activation of microglia and astrocytes, is a significant component of the pathophysiological process of HIE [8,9,10].

Concomitant with the established role of inflammation in the etiology of HIE, there is emerging interest in hematological indices as markers of systemic inflammation and possible prognostic indicators. These indices, including the neutrophil-to-lymphocyte ratio (NLR), platelet-to-lymphocyte ratio (PLR), and systemic immune-inflammation index (SII), have been previously employed as prognostic markers for various adult medical conditions, including malignancies [11,12]. Notably, recent studies have begun to examine a novel index, termed the hemoglobin, albumin, lymphocyte, and platelet (HALP) scores, for its potential to reflect both nutritional status and systemic inflammation [11,12]. These indices offer the advantage of being calculated from low-cost routine measures in the neonatal intensive care unit (NICU), making them potentially valuable additions to the existing diagnostic criteria.

Given the multifaceted role of inflammation in the pathology of HIE, this study aimed to elucidate the diagnostic and prognostic utility of systemic inflammatory indices and HALP scores in infants diagnosed with moderate-to-severe HIE. We will further investigate the modulatory effects of therapeutic hypothermia (HT) treatment on these parameters. Additionally, we aimed to determine the association between systemic inflammatory indices, HALP scores, and key clinical outcomes, including renal failure, seizures, periventricular leukomalacia (PVL), intraventricular hemorrhage (IVH), and imaging findings in the HIE cohort.

The novelty of our research lies in the incorporation of a broad range of systemic inflammatory indices, some of which, such as the HALP score, are novel in the context of HIE, thereby bridging the gap in the existing literature. Additionally, to our knowledge, the modulation of these indices by HT treatment has not been adequately investigated, offering an avenue for further research and therapeutic intervention.

## 2. Material and Methods

### 2.1. Overview and Ethical Consideration

This retrospective cohort study was conducted to investigate systemic inflammatory indices and HALP scores among neonates diagnosed with hypoxic ischemic encephalopathy (HIE) and to examine the impact of hypothermia therapy (HT) on newborns with moderate-to-severe hypoxic–ischemic encephalopathy (HIE). This study was conducted in the tertiary-level neonatal intensive care unit (NICU) at Marmara University, Faculty of Medicine, Department of Neonatology, between June 2023 and August 2023, and the data of patients hospitalized between 2014 and March 2022 were compiled. Ethical approval for this study was obtained from the Local Ethics Committee of Marmara University, aligning with the ethical principles delineated by the Declaration of Helsinki; numbered: 09.2023.823, dated: 2 June 2023.

### 2.2. Study Population and Inclusion/Exclusion Criteria

The participants were 43 neonates who were ≥36 weeks of gestation and displayed indications of moderate-to-severe HIE. The inclusion criteria were a 10 min Apgar score ≤ 5, arterial pH < 7, base deficit > −16, or ongoing resuscitation at 10 min. To validate the moderate-to-severe HIE diagnosis and initiate the HT protol, Thompson, Çakır, Sarnat and Sarnat staging [13,14,15] within 6 h post-birth or specific abnormal patterns on amplitude-integrated electroencephalography (aEEG) were considered.

The control cohort included neonates with a gestational age of at least 36 weeks, all of whom were free from hypoxic–ischemic encephalopathy, sepsis, chromosomal abnormalities, and congenital diseases. Neonates diagnosed with transient tachypnea and monitored in the NICU were chosen to ensure a homogenous group by excluding neonates born to mothers with chronic or gestational conditions. Thus, the control group specifically comprised non-outpatient infants without widespread health issues to eliminate confounding variables and enhance the study’s methodological rigor.

### 2.3. Treatment Protocol

Hypothermia therapy (HT) was implemented in the intervention group using a standardized whole-body cooling protocol [16]. The decision to initiate HT was predominantly based on Sarnat and Sarnat staging [13] to determine the severity of HIE as stage 1–3 (mild, moderate, and severe) and to ascertain eligibility for hypothermia treatment (HT) [14]. Additionally, patients presenting with comorbidities, such as sepsis, initial liver or renal injury, or significant arrhythmias were not included, ensuring a homogeneous patient cohort with no divergent treatment protocols.

### 2.4. Data Collection and Variables

The study data were compiled retrospectively between June 2023 and August 2023 at the Department of Neonatology, Marmara University. Clinical, demographic, and laboratory data were collected retrospectively from medical records, necessitating meticulous care to minimize errors and biases. Inflammatory indices were computed based on peripheral whole-blood cell counts acquired (before the initiation of HT) within the first 0–6 h post-birth and during hours 60–72 post-birth (that is during HT therapy or toward completing hypothermia therapy).

These indices are pivotal tools for assessing systemic inflammation and have been employed in previous HIE studies as follows:

NLR (neutrophil-to-lymphocyte ratio): N/L.
PLR (platelet-to-lymphocyte ratio), p/L.
Monocyte-to-lymphocyte ratio (MLR) (M/L).
Systemic immune-inflammation index (SII): P × N/L.
SIRI (systemic inflammation response index): N × M/L.
PIV (platelet, neutrophil, monocyte, and lymphocyte index): P × N × M/L.
HALP score: hemoglobin (g/L) × albumin (g/L) levels × lymphocyte count (/L)/platelet count (/L).

### 2.5. Statistical Analysis

Systemic inflammatory indices were analyzed before and post-HT in the HIE group. This study also probed the differences in these markers between the HIE and control groups within the first 12 h and last 60–72 h. Correlations between renal failure, seizure presence, aEEG abnormalities, imaging findings, and inflammatory indices in the HIE group were meticulously examined.

IBM SPSS Statistics version 22.0 was deployed for statistical evaluations. A repertoire of tests, including Kolmogorov–Smirnov, Mann–Whitney U, Chi-Squared, and Wilcoxon rank tests, were applied for the comparative analysis of demographic variables, systemic inflammatory markers, and other pertinent parameters.

## 3. Results

### 3.1. Analysis of Clinical and Demographic Parameters in HIE and Control Cohorts

A total of 43 HIE patients and 50 controls were included in the study. The clinical and demographic parameters revealed significant differences in APGAR scores at 1 and 5 min, cord blood pH, and base excess between the HIE and control groups (*p* < 0.001). No substantial disparity was observed in gestational weeks (GW) or birth weights (BW) between the groups. Variables such as cesarean section (C/S), female sex, and cord blood lactate levels are illustrated in Table 1, providing a comprehensive view of the clinical and demographic characteristics of both cohorts.

### 3.2. Temporal Changes and Associations in Systemic Inflammatory Markers and Hematological Parameters in Hypoxic–Ischemic Encephalopathy (HIE) and Control Groups

Significant differences were observed in multiple hematological and systemic inflammatory parameters between the HIE and control groups. Hemoglobin and albumin levels were notably lower in the HIE cohort at both the 0–6 and 60–72 h time points (*p* < 0.001). Neutrophil counts remained statistically similar between the groups, while lymphocyte counts were elevated in the HIE group at 0–6 h (*p* = 0.001). Monocyte and platelet levels were significantly reduced in the HIE group at both time points (*p* < 0.001). Indices of systemic inflammation, such as SII, PIV, SIRI, and HALP scores, displayed marked elevations in the HIE group, particularly before TH initiation and at 0–6 h, compared to 60–72-h’ measures. The neutrophil-to-lymphocyte ratio was lower in the HIE group at 60–72 h compared the control group (*p* = 0.002), and variations in the platelet-to-lymphocyte ratio were statistically significant and lower than those in the control group, especially in the 0–6-h time frame (*p* < 0.001) (Table 2.)

### 3.3. Comparative Analysis of Temporal Variations in Hematological and Systemic Inflammatory Parameters before the Therapeutic Hypothermia Treatment and during/or toward Finishing the Hypothermia Therapy in HIE Patients

This study delineated the comparative temporal variations in hematological and systemic inflammatory markers, including the HALP score, in patients with HIE before and after undergoing therapeutic hypothermia, revealing various parameter alterations between two critical time points, 0–6 h and 60–72 h, during and post-HT treatment.

Hemoglobin levels experienced a discernible alteration, reducing from 17.00 g/dL at 0–6 h to 14.90 g/dL at 60–72 h (*p* = 0.003). Albumin levels underwent a decline from 3.50 g/dL to 3.25 g/dL (*p* = 0.005).

Conversely, neutrophil counts decreased from 11,100 cells/μL to 5500 cells/μL (*p* < 0.001), and lymphocyte counts decreased from 5900 cells/μL to 2300 cells/μL (*p* < 0.001). Monocyte counts decreased from 1000 to 400 cells/μL (*p* < 0.001). Platelet counts followed a similar trend, decreasing from 217,000 to 141,000 cells/μL (*p* < 0.001). Furthermore, the white blood cell count notably decreased from 18,200 cells/μL to 10,100 cells/μL (*p* < 0.001). The neutrophil-to-lymphocyte ratio showed a significant increase from 1.53 to 2.79 (*p* = 0.019), and the platelet-to-lymphocyte ratio (PLT/L) escalated from 33.24 to 51.00 (*p* = 0.002).

The PIV and HALP scores demonstrated a considerable reduction (351.54 to 124.03 and 181.09 to 99.39, respectively) (*p* < 0.001). Table 3 shows the temporal variations in each parameter before and during therapy or upon completion of HT treatment.

### 3.4. Analysis of Hematological and Inflammatory Markers in HIE Patients according to Clinical Subgroups

This analysis aimed to discern variations in inflammatory markers and HALP scores in patients with HIE, with a specific focus on delineating differences according to clinical subgroups: the presence of seizures, intraventricular hemorrhage (IVH), periventricular leukomalacia (PVL), and kidney injury.

A significant variance in the HALP score was observed at 0–6 h between patients who experienced seizures and those who did not (*p* = 0.022). In the IVH group, the SIRI scores at 60–72 h were significantly higher than those in patients without IVH (*p* = 0.041). Although not significantly different, the HALP score at 0–6 h was higher in patients with IVH (168 vs. 181). Similarly, the HALP score at 0–6 h exhibited a substantial difference and elevation between patients with and without PVL (*p* = 0.004), indicating a distinct hematological response in these groups. Markers, including PLT indexes at 0–6 and 60–72 h, NLR at 60–72 h, PLT/L at 0–6 h, and HALP score at 0–6 h were significantly elevated in patients with renal injury (Table 4).

### 3.5. Predictive Value of Inflammatory Markers and HALP Score in HIE Patients

The efficacy of inflammatory markers and the HALP Score in predicting the clinical trajectories of patients with hypoxic–ischemic encephalopathy (HIE) from the time of hospital admission was analyzed. These findings illustrate that NLR, PLR, MLR, SII, and SIRI have significant prognostic value, with MLR emerging as the most predictive marker of clinical morbidity outcomes (including seizures, IVH, PVL, and kidney injury) in patients with HIE. Conversely, the HALP and PIV scores exhibited lower predictive reliability.

Each marker was analyzed for sensitivity, specificity, and area under the curve (AUC) to assess its discriminative ability, with significant *p*-values indicating robust predictive potential. MLR demonstrated high sensitivity and specificity (0.707 and 0.718, respectively) and a substantial AUC of 0.766, reinforcing the potent predictive capability of this marker to predict outcomes, including seizures, IVH, PVL, and kidney injury in patients with HIE.

**Figure 1:**
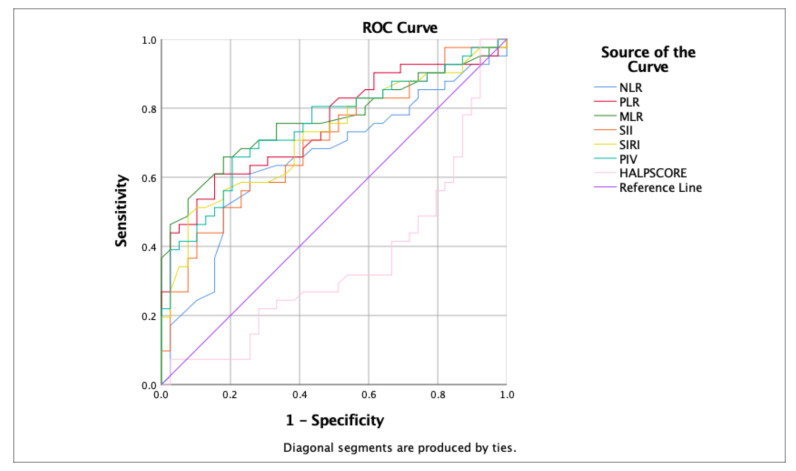
Comparative ROC curve analyses of hematological and inflammatory markers for predicting clinical outcomes in hypoxic–ischemic encephalopathy (HIE) patients.

Table 5 presents a comprehensive statistical analysis of each marker, and Figure 1 provides comparative ROC curve analyses, elucidating the differential predictive capacities of these hematological and inflammatory markers in patients with HIE.

## 4. Discussion

Advancements in neonatology and neurology have highlighted the significant role of inflammation as a secondary yet powerful initiator of neonatal brain injuries.

This study investigated the dynamic interplay between hematological and inflammatory markers in patients diagnosed with hypoxic–ischemic encephalopathy (HIE) undergoing therapeutic hypothermia treatment.

Hypoxic–ischemic encephalopathy (HIE) is associated with a significant inflammatory response that has been linked to worsened brain damage following ischemia. A pivotal study in this field was conducted by Ceran et al., who assessed the diagnostic role of systemic inflammatory indices in infants with moderate-to-severe HIE [11]. This study compared the systemic inflammatory indices between infants with HIE and healthy controls.

Upon a detailed review of HIE versus control data, our study revealed differences in inflammatory markers, particularly during and post-HT; that is at 60–72 h. Markers such as the neutrophil-to-lymphocyte ratio (NLR) and the monocyte-to-lymphocyte ratio (MLR) were notably lower in the HIE group following hypothermia therapy, potentially indicating suppression of the inflammatory surge typically observed in these neonates. Evidence suggests that hypothermia treatment can reduce inflammation. Studies have shown that hypothermia exerts neuroprotective effects in peripheral nerve ischemia by attenuating the inflammatory response. This effect was observed through the reduction in key inflammatory mediators, such as cytokines, ICAM-1, and NF-kappa B, as well as a decrease in granulocyte and mononuclear phagocyte infiltration into nerve tissue with intraischemic hypothermia. This suggests that therapeutic hypothermia can modulate the inflammatory response after ischemic injury [17,18]. This suppression may indicate the influence of HT in modulating the inflammatory response, a finding that merits further investigation and discussion in this field. The variation in our results may in part stem from the composition of our control group, which included newborns diagnosed with transient tachypnea (TTN). This condition may have introduced additional variability and a higher baseline level of inflammation, thereby impacting the comparability of the study with the HIE group. We did not structure the study to compare the systemic inflammatory indices between exclusively mild-to-severe HIE groups either; the outcomes might have been more distinct and more remarkable then. The inclusion of TTN patients in the control group could be considered a significant limitation of our study design.

Upon analyzing the temporal variations in hematological and inflammatory markers among patients with HIE, our findings delineate significant shifts between two time points: the time before HT and at 60–72 h during the therapy/or toward the end of the hypothermia therapy. These shifts suggest marked responses to hypothermia treatment and provide insights into the dynamic nature of HIE progression and the response to intervention. Hemoglobin and albumin levels showed notable decreases after HT, suggesting a potential compensatory mechanism after hypoxic events. Albumin may act as a positive acute-phase protein after HT. Noteworthy declines in hemoglobin and albumin levels post-HT suggest a possible physiological adaptation to hypoxic stress. The increase in white blood cell counts, including neutrophils, lymphocytes, and monocytes, further underscores the ongoing systemic inflammation in HIE, which may mitigate HT [19,20].

The clinical importance of these variations needs to be clarified, and neutrophil infiltration at early stages is crucial for exacerbating brain injury in newborns caused by hypoxia–ischemia, a condition in which inflammation heightens brain damage [19]. The entry and activity of monocytes in the ischemic brain, particularly under conditions such as hypoxic–ischemic encephalopathy (HIE) and neonatal arterial ischemic stroke (NAIS), are key areas of focus [19,20]. Understanding how these cells contribute to brain injury may pave the way for innovative therapeutic approaches. Strategies that target monocyte activity might offer new treatment avenues, and harnessing monocytes for cell-based therapies holds potential as a future treatment modality. In this study, the conspicuous surge in neutrophil, lymphocyte, and monocyte counts between the two intervals could be attributed to a heightened inflammatory response, corroborating the finding that neuroinflammation is a vital contributor to the HIE pathophysiology [6]. Elevated WBC counts further accentuate this inference, emphasizing a heightened systemic inflammatory response post-hypoxia. The neutrophil/lymphocyte ratio, a prominent systemic inflammatory marker, showed significant shifts [17,21,22,23], echoing the findings of several studies that validated its prognostic utility in various conditions.

Albumin, a critical serum protein, also shows notable variations [2], suggesting alterations in protein synthesis or degradation after treatment. According to prior research, hemoglobin plays a crucial role in neonatal hypoxic responses [19,20,24], and its elevation can be perceived as an adaptive response to hypoxia.

The only study that evaluated systemic inflammatory indices in infants with moderate-to-severe Hypoxic–ischemic encephalopathy reported that an elevated NLR level > 1.12 was found to be an independent predictor for HIE [11]. No associations were found between systemic inflammatory indices and amplitude-integrated electroencephalography (aEEG) patterns, the presence of seizures, or death. In a study by Ceran et al., the predictive capabilities for HIE, as determined by the area under the curve for NLR, PLR, MLR, SII, SIRI, and PIV, were significant (0.808, 0.597, 0.653, 0.763, 0.686, and 0.663, respectively) [11]. These results are consistent with those of the present study. In our study, we determined that NLR > 2 was a significant predictor of HIE severity. To the best of our knowledge, for the first time in the literature, we have determined significant predictive values to predict KIE morbidities with the following values: PLR measured at 54.75, MLR measured at 0.305, SII measured at 458.35, SIRI measured at 2.7, PIV measured at 603.5, and HALP Score measured at 140.7.

These modest variations in MPV may indicate a nuanced alteration in platelet production or degradation after hypoxia. Further studies are required to clarify these findings with a broader understanding of neuroinflammation [5,6,7], neuroimmunometabolism [14,15,25], and the intricate interplay between systemic inflammatory responses [8,9,10,11,12].

Markers like MLR, SII, and SIRI showed mixed responses, with some portraying evident variations and others, such as MLR and SII, reflecting subtler shifts [3,21,23,24]. Alterations in these markers merit further exploration given their touted significance as inflammatory and immune responses. To the best of our knowledge, the present study is the second to investigate systemic inflammation in HIE patients. A previous study by Ceran et al. reported that infants with HIE displayed notably elevated NLR, SII, PIV, and SIRI values compared with their control counterparts, with statistical significance of *p* = 0.001 for the first three and *p* = 0.004 for SIRI. After hypothermia treatment, these metrics showed a marked decline in the HIE group [9].

According to our study, which is the first to investigate HALP scores in infants with HIE, particular attention was given to the HALP score, which exhibited a significant increase between two-time intervals: before and at the end of HT. Notably, the HALP score at 0–6 h exhibited a significant elevation in the initial hours of life in HIE patients who experienced seizures, PVL, and kidney injury. This underscores the predictive capacity of the HALP score for these specific morbidities in HIE patients.

A consistent pattern of significant differences was particularly noticeable in patients with renal injury, where markers such as PLT index at 0–6 h and 60–72 h, NLR at 60–72 h, PLT/L at 0–6 h, and SII at 0–6 h were significantly elevated in the presence of renal impairment. Similarly, a prominent elevation in the HALP score at 0–6 h was discerned in cases with PVL, establishing a clear link between elevated early HALP scores and the likelihood of developing PVL.

We present that the prognostic significance of early HALP scores in forecasting specific morbidities such as PVL, seizures, and renal injury in patients with HIE is of paramount importance. This early predictive capability is vital for facilitating timely and targeted clinical interventions, potentially altering the course of these morbidities. However, the broader implications of variations in other systemic inflammatory markers remain to be fully understood. A comprehensive investigation into these variations is essential to unravel their complete predictive and prognostic value in relation to the other morbidities associated with HIE. Such an understanding is crucial for enhancing patient outcomes and refining therapeutic strategies in neonatal care.

The results of this investigation suggest a role for hematological and inflammatory markers in predicting clinical outcomes in patients with HIE. These markers may have diagnostic and prognostic utility for managing patients with HIE.

The variability in the cut-off values of markers such as NLR, PLR, and MLR across different clinics underscores the importance of contextual interpretation of these markers [20,21,24]. In our study, MLR demonstrated the most substantial predictive capability, suggesting its potential role as an efficacious tool in predicting critical clinical outcomes, facilitating early and precise interventions, and improving clinical outcomes in HIE patients.

The variance in the interpretative values of markers among different clinical pictures signifies the importance of developing standardized cut-off values for universal applicability, ensuring accuracy and reliability in clinical settings [22,23,26].

Future investigations should aim to corroborate the findings of this study, determine more specific cut-off values, and explore the longitudinal variations and interactions of these markers to gain a more comprehensive understanding. This would potentially optimize the management of and treatment strategies for patients with HIE, allowing for more individualized and effective approaches and advancing the fields of neurology and neonatal care.

### 4.1. Study Strengths

This study stands out for its multifaceted analytical approach that provides intricate and nuanced insights into physiological and pathological alterations associated with HIE in patients undergoing therapeutic hypothermia treatment. It meticulously evaluates temporal variations in hematological and inflammatory markers at pretreatment and treatment time intervals, offering a deeper understanding of evolving physiological states. A particularly noteworthy contribution of this study is its innovation in prognostic markers to identify and validate advanced indicators, such as SIRI, SII, PIV, and HALP scores in the context of HIE, paving new pathways for assessing disease progression and treatment response. These innovations significantly elevate the clinical understanding of these markers in predicting HIE patient outcomes, thereby facilitating the development of more personalized and precise intervention strategies and enhancing patient care and prognosis. To the best of our knowledge, the present study is the first to investigate systemic inflammatory indices and HALP scores in the HİE cohort.

### 4.2. Study Limitations

Sample Size: Our research relies on a finite and specific number of HIE patients and controls, potentially limiting the breadth of our insights. To better understand and generalize our findings, future studies should include a larger and more diverse group of participants.

Control Group: The absence of healthy neonates in the control group inhibits our understanding of how systemic inflammatory markers differ compared with healthy controls. Reconducting this study to stage 1 mild HIE cases as a control group could potentially enhance the overall value and insights derived from this study, offering a refined perspective for future research.

Single-Center Study: this study collected and analyzed data exclusively from one clinical environment, implying that the findings might not be universally applicable because of the diverse clinical practices and patient demographics in different healthcare settings.

Temporal Analysis: This study assessed temporal variations in markers after the application of therapeutic hypothermia treatment but did not extend to long-term follow-up. A more prolonged observation period would elucidate the lasting effects and predictive efficacy of the studied markers and contribute to a more profound understanding of their clinical significance.

Cut-off Variability: This study observed variability in the established cut-off values for markers such as NLR, PLR, and MLR across different clinics, signifying the possibility of regional or population-specific differences. This variability underscores the importance of conducting broader validation studies to confirm the applicability and reliability of these cut-off values across diverse settings and populations.

## 5. Conclusions

This research has profoundly emphasized the potential of hematological and inflammatory markers, with a particular emphasis on MLR, in presenting themselves as state-of-the-art predictive tools for HIE patients. Their integration into clinical assessments may offer a platform to initiate early and tailored interventions.

We have demonstrated that SIRI, SII, PIV, and HALP scores are pioneering and easily accessible indicators for assessing the progression of hypoxic–ischemic encephalopathy (HIE) and its associated responses to hypothermia therapy. Further large-scale studies are needed to enhance our understanding of HIE progression so that treatment modalities may be better tailored to individual patient needs.

## Figures and Tables

**Table 1:** Clinical and demographic characteristics of the HIE and control groups.

Variable	HIE (n = 43)	Control (n = 50)	*p*-Value
Cesarean Section (C/S). n (%)	27 (62.8%)	39 (78.0%)	0.117
Female Gender. n (%)	25 (58.1%)	25 (50.0%)	0.532
Gestational Weeks (GW). mean ± sd	38.19 ± 2.06	37.60 ± 1.65	0.132
Birth Weight (BW). median. IQR	3295.00. 2720.00–3565.00	3110.00. 2657.50–3445.00	0.339
APGAR1 Score. median. IQR	3.00. 1.00–4.75	8.00. 7.00–9.00	<0.001
APGAR5 Score. median. IQR	5.00. 3.00–7.00	9.00. 8.00–10.00	<0.001
Cord Blood pH. mean ± sd	6.82 ± 0.16	7.25 ± 0.09	<0.001
Base Deficit. median. IQR	−18.90. −23.80 to −17.00	−5.00. −7.70 to −3.18	<0.001
Cord Blood Lactate. median. IQR	16.00. 11.10–18.00	3.00. 2.45–4.35	<0.001

*p*: independent *t*-test. Mann–Whitney U Test. HIE: Hypoxic–Ischemic Encephalopathy; APGAR: Appearance (skin color); Pulse (heart rate); Grimace Response (reflexes); Activity (muscle tone); Respiration (breathing rate and effort); IQR: Interquartile Range.

**Table 2:** Comparative analysis of hematological and systemic inflammatory parameters in HIE and control groups at 0–6 and 60–72 h.

	HIE (n = 43)			Control (n = 50)			
	Median	IQR		Median	IQR		*p*
Hb 0–6	17.00	15.60	18.10	18.90	17.08	19.83	<0.001
Hb 60–72	14.90	12.35	17.65	17.40	15.85	18.40	0.004
Alb 0–6	3.50	3.05	3.90	3.70	3.50	3.90	0.163
Alb 60–72	3.25	2.80	3.50	3.70	3.40	4.00	<0.001
Neu 0–6	11,100.00	7100.00	15,200.00	10,150.00	7300.00	14,500.00	0.844
Neu 60–72	5500.00	3350.00	8650.00	5000.00	3850.00	7300.00	0.796
L 0–6	5900.00	3900.00	9400.00	3800.00	3200.00	5375.00	0.001
L 60–72	2300.00	1550.00	3250.00	3200.00	2850.00	4400.00	0.001
M 0–6	1000.00	600.00	1800.00	1600.00	1100.00	2000.00	0.004
M 60–72	400.00	200.00	800.00	1200.00	950.00	1450.00	<0.001
MPV 0–6	7.95	7.28	9.25	10.15	9.58	10.73	<0.001
MPV 60–72	8.30	7.55	9.95	10.40	9.70	11.10	<0.001
PLT 0–6	217,000.00	137,000.00	260,000.00	252,000.00	187,500.00	299,750.00	0.018
PLT 60–72	141,000.00	68,500.00	187,500.00	248,000.00	175,000.00	322,500.00	<0.001
WBC 0–6	18,200.00	14,200.00	25,300.00	17,450.00	13,975.00	20,875.00	0.155
WBC 60–72	10,100.00	6250.00	12,400.00	10,200.00	8500.00	12,850.00	0.245
PLT index 0–6	1638.00	1132.20	2109.70	2539.80	2002.20	2967.50	<0.001
PLT index 60–72	1.099.80	681.60	1585.40	2551.10	1856.70	3351.75	<0.001
NLR 0–6	1.53	1.04	3.67	2.64	1.64	4.15	0.021
NLR 60–72	2.79	1.47	4.85	1.68	1.21	2.10	0.002
PLT/L 0–6	33.24	19.39	61.30	58.40	43.14	80.24	<0.001
PLT/L 60–72	51.00	41.51	91.52	64.59	50.17	94.22	0.108
MLR 0–6	0.18	0.09	0.38	0.38	0.25	0.53	<0.001
MLR 60–72	0.22	0.09	0.40	0.33	0.29	0.39	0.021
SII 0–6	331.02	161.17	592.00	548.68	361.20	1002.61	0.004
SII 60–72	327.13	175.15	681.52	340.00	240.18	583.28	0.485
PIV 0–6	351.54	111.31	729.64	816.93	531.11	1925.78	<0.001
PIV 60–72	124.03	36.43	461.54	462.04	230.16	607.67	0.001
SIRI 0–6	1.69	0.69	4.66	3.82	2.00	6.84	0.001
SIRI 60–72	0.85	0.42	2.76	1.89	1.27	2.62	0.062
HALP score 0–6	181.09	96.94	283.01	112.96	75.86	173.71	0.02
HALP score 60–72	99.39	51.15	138.61	95.62	69.22	132.67	0.382

HIE—Hypoxic–Ischemic Encephalopathy; Hb—Hemoglobin; Alb—Albumin; Neu—Neutrophils; L—Lymphocytes; M—Monocytes; MPV—Mean Platelet Volume; PLT—Platelets; WBC—White Blood Cell Count; NLR—Neutrophil-to-Lymphocyte Ratio; MLR—Monocyte-to-Lymphocyte Ratio; SII—Systemic Immune-Inflammation Index; PIV—Platelet, Neutrophil, Monocyte, and Lymphocyte Index; SIRI—Systemic Inflammation Response Index; HALP—Hemoglobin, Albumin, Lymphocyte, and Platelet Score; IQR, Interquartile Range. *p*—Mann–Whitney U test.

**Table 3:** Temporal variation of hematological and inflammatory markers in HIE patients at two different time points before, during, and after therapeutic hypothermia treatment (0–6 h and 60–72 h).

HIE (n = 43)							
Parameter	0–6 h			60–72 h			
	Median	IQR		Median	IQR		*p*
Hb (g/dL)	17.00	15.60	18.10	14.90	12.35	17.65	0.003
Alb (g/dL)	3.50	3.05	3.90	3.25	2.80	3.50	0.005
Neu (cells/μL)	11,100.00	7100.00	15,200.00	5500.00	3350.00	8650.00	<0.001
L (cells/μL)	5900.00	3900.00	9400.00	2300.00	1550.00	3250.00	<0.001
M (cells/μL)	1000.00	600.00	1800.00	400.00	200.00	800.00	<0.001
MPV	7.95	7.28	9.25	8.30	7.55	9.95	0.221
PLT (cells/μL)	217,000.00	137,000.00	26,000.00	141,000.00	68,500.00	187,500.00	<0.001
WBC (cells/μL)	18,200.00	14,200.00	25,300.00	10,100.00	6250.00	12,400.00	<0.001
PLT Index	1638.00	1132.20	2109.70	1099.80	681.60	1585.40	<0.001
NLR	1.53	1.04	3.67	2.79	1.47	4.85	0.019
PLT/L	33.24	19.39	61.30	51.00	41.51	91.52	0.002
MLR	0.18	0.09	0.38	0.22	0.09	0.40	0.166
SII	331.02	161.17	592.00	327.13	175.15	681.52	0.908
PIV	351.54	111.31	729.64	124.03	36.43	461.54	0.048
SIRI	1.69	0.69	4.66	0.85	0.42	2.76	0.246
HALP Score	181.09	96.94	283.01	99.39	51.15	138.61	<0.001

HIE: Hypoxic–Ischemic Encephalopathy; IQR: Interquartile Range; Hb: Hemoglobin; Alb: Albumin; Neu: Neutrophils; L: Lymphocytes; M: Monocytes; MPV: Mean Platelet Volume; PLT: Platelets; WBC: White Blood Cell Count; PLT Index: Platelet Index; NLR: Neutrophil-to-Lymphocyte Ratio; PLT/Lenf: Platelet-to-Lymphocyte Ratio; MLR: Monocyte-to-Lymphocyte Ratio; SII: Systemic Immune-Inflammation Index; PIV: Platelet Inflammation Value; SIRI: Systemic Inflammation Response Index; HALP Score: Hypoxia-Associated Lethal Pathophysiology Score. *p*: *p*-value calculated using the Wilcoxon signed-rank test.

**Table 4:** Comparison of hematological and inflammatory markers according to clinical outcomes in patients with HIE.

HIE (n = 43)			
	Seizures		
	No (n = 21)	Yes (n = 22)	*p*
	Median (IQR)	Median (IQR)	
PLT index 0–6	1743 (1122.25–2157.15)	1511.6 (1126.58–2186.35)	0.706
PLT index 60–72	1099.8 (805.6–1630)	1209.3 (613.55–1605.05)	0.885
NLR 0–6	1.74 (1.24–3.91)	1.45 (0.74–3.21)	0.325
NLR 60–72	2.56 (1.46–4.07)	3.52 (1.46–5.19)	0.270
PLT/LENF 0–6	49.25 (29.23–63.4)	22.45 (14–50.01)	0.055
PLT/LENF 60–72	44.05 (42.19–65.68)	55.42 (39.31–100.96)	0.278
MLR 0–6	0.23 (0.1–0.41)	0.12 (0.09–0.23)	0.142
MLR6 0–72	0.16 (0.08–0.38)	0.25 (0.1–0.47)	0.406
SII 0–6	473.12 (205.52–795.48)	300.22 (130.82–507.11)	0.285
SII 60–72	257.39 (170.63–442.43)	382.38 (173.3–725.32)	0.406
PIV 0–6	385.5 (113.56–1267.83)	274.2 (100.21–665.48)	0.528
PIV 60–72	124.03 (34.13–216.73)	213.66 (36.65–561.91)	0.492
SIRI 0–6	1.89 (0.72–5.52)	1.31 (0.58–3.33)	0.466
SIRI 60–72	0.65 (0.4–1.58)	1.46 (0.56–3.5)	0.214
HALP score 0–6	130.5 (85.94–200.65)	251.85 (152.55–443.99)	0.022
HALP score 60–72	110.89 (72.23–173.51)	58.68 (42.2–128.83)	0.160
	IVH		
	No (n = 35)	Yes (n = 7)	*p*
	Median (IQR)	Median (IQR)	
PLT index 0–6	1743 (1201.2–2512.3)	1207.8 (908.8–2024)	0.133
PLT index 60–72	1138.2 (685.8–1563.1)	1277.4 (414.15–1956.05)	0.772
NLR 0–6	1.49 (1.04–3.54)	1.75 (0.77–6.94)	0.353
NLR 60–72	2.74 (1.38–4.19)	4.25 (1.16–11.01)	0.359
PLT/LENF 0–6	33.24 (20.44–61.19)	32.11 (9.04–118.13)	0.933
PLT/LENF 60–72	49.88 (40.32–91.2)	73.84 (35.97–116.52)	0.469
MLR 0–6	0.18 (0.08–0.38)	0.13 (0.11–0.23)	0.826
MLR 60–72	0.19 (0.09–0.39)	0.27 (0.13–0.66)	0.530
SII 0–6	317.71 (161.17–569.25)	362.67 (79.51–1311.19)	0.853
SII 60–72	269.69 (166.52–460.43)	520.91 (142.23–1114.03)	0.385
PIV 0–6	385.5 (111.75–729.64)	262.24 (42–544)	0.489
PIV 60–72	94.97 (35.53–325.08)	377.52 (41.17–584.82)	0.359
SIRI 0–6	1.75 (0.69–4.66)	1.39 (0.6–4.25)	0.748
SIRI 60–72	0.69 (0.37–1.81)	2.89 (1–8.47)	0.041
HALPSCORE 012	168.03 (104.22–283.01)	181.09 (49.17–589.54)	0.735
HALPSCORE 60–72	101.35 (53.15–148.13)	81.55 (38.65–148.89)	0.653
	PVL		
	No (n = 29)	Yes (n = 10)	*p*
	Median (IQR)	Median (IQR)	
PLT index 0–6	1743 (1215.7–2367.7)	1325.6 (973.08–2140.08)	0.260
PLT index 60–72	1138.2 (650.25–1810.35)	1399.75 (494.25–1607.7)	0.925
NLR 0–6	1.69 (1.24–3.91)	1.22 (0.73–3.46)	0.288
NLR 60–72	2.79 (1.48–4.6)	2.62 (1.06–8.22)	0.851
PLT/LENF 0–6	49.25 (24.21–64.19)	19.92 (11.45–40.65)	0.022
PLT/LENF 60–72	53.18 (40.26–100.96)	46.03 (41.17–88.65)	0.639
MLR 0–6	0.18 (0.08–0.35)	0.22 (0.13–0.47)	0.234
MLR 60–72	0.19 (0.09–0.35)	0.27 (0.09–0.65)	0.336
SII 0–6	439.42 (206–795.48)	289.42 (98.05–502.84)	0.234
SII 60–72	318.43 (177.66–538.61)	253.4 (107.49–756.95)	0.778
PIV 0–6	309.79 (121.27–654.07)	638.96 (105.45–940.54)	0.260
PIV 60–72	115.99 (35.53–325.08)	280.15 (28.01–665.49)	0.482
SIRI 0–6	1.5 (0.59–3.96)	2.78 (1–4.96)	0.234
SIRI 60–72	0.78 (0.4–2.08)	1.1 (0.23–3.66)	0.838
HALP core 0–6	136.19 (84.4–238.98)	327.32 (218.88–583.45)	0.004
HALP core 60–72	109.68 (47.21–135.54)	88.22 (48.41–150.02)	0.884
	Kidney Injury		
	No (n = 28)	Yes (n = 15)	
	Median (IQR)	Median (IQR)	*p*
PLT index 0–6	230,000 (185,000–228,350)	137,000 (117,000–218,000)	0.006
PLT index 60–72	169,500 (114,000–207,250)	92,000 (50,000–139,500)	0.013
NLR 0–6	1.70 (1.25–4.27)	1.20 (0.6–1.8)	0.083
NLR 60–72	2.45 (1.2–3.85)	4.1 (2.7–6.25)	0.022
PLT/L 0–6	53.35 (23.85–64.62)	20.8 (11.9–32.1)	0.004
PLT/L 60–72	58.7 (43.65–97.67)	42.40 (33.80–69.15)	0.173
MLR 0–6	0.21 (0.1–0.40)	0.11 (0.04–0.23)	0.092
MLR 60–72	0.19 (0.08–0.35)	0.25 (0.13–0.63)	0.217
SII 0–6	475 (242–801)	199 (79.5–334.6)	0.012
SII 60–72	304 (174–624)	354 (159–717)	0.941
PIV 0–6	438 (226–821)	111 (39.4–544)	0.037
PIV 60–72	125 (34.8–311)	82 (42.5–625)	0.73
SIRI 0–6	2.25 (1.05–5.07)	0.8 (0.3–4.3)	0.12
SIRI 60–72	0.8 (0.35–1.8)	1.1 (0.5–7.3)	0.126
HALP score 0–6	124 (83.5–265)	245 (200–566)	0.003
HALP score 60–72	109 (47.6–129)	97.4 (52.8–148)	0.985

Neutrophils; Lenf: Lymphocytes; Monosit: Monocytes; PLT: Platelets; PLT Index: Platelet Index; NLR: Neutrophil-to-Lymphocyte Ratio; PLT/Lenf: Platelet-to-Lymphocyte Ratio; MLR: Monocyte-to-Lymphocyte Ratio; SII: Systemic Immune-Inflammation Index; PIV: Platelet Inflammation Value; SIRI: Systemic Inflammation Response Index; HALP Score: Hypoxia-Associated Lethal Pathophysiology Score; IVH, intraventricular hemorrhage; PVL, periventricular leukomalacia. *p*: Mann–Whitney U test.

**Table 5:** Predictive efficacy of hematological and inflammatory markers for severe clinical outcomes in patients with HIE.

Marker	Cut-Off Value	Sensitivity	Specificity	Area under the Curve (AUC)	Lower 95% CI	Upper 95% CI	*p*
NLR	2	0.634	0.641	0.654	0.532	0.776	0.018
PLR	54.75	0.659	0.643	0.751	0.644	0.859	0.001
MLR	0.305	0.707	0.718	0.766	0.659	0.873	0.001
SII	458.35	0.634	0.641	0.700	0.585	0.814	0.002
SIRI	2.7	0.634	0.615	0.722	0.61	0.834	0.001
PIV	603.5	0.707	0. 723	0.749	0.641	0.857	0.001
HALP Score	140.7	0.39	0.333	0.349	0.226	0.472	0.020

SII: Systemic Immune-Inflammation Index; PIV: Platelet Inflammation Value; SIRI: Systemic Inflammation Response Index; HALP Score: Hypoxia-Associated Lethal Pathophysiology Score.

## Data Availability

The data presented in this study are available on request from the corresponding author. The data are not publicly available due to patients’ and hospital privacy.
